# Algorithm for sample availability prediction in a hospital-based epidemiological study spreadsheet-based sample availability calculator

**DOI:** 10.1038/s41598-021-03399-1

**Published:** 2022-02-03

**Authors:** Amrit Sudershan, Kanak Mahajan, Rakesh K. Panjaliya, Manoj K. Dhar, Parvinder Kumar

**Affiliations:** 1 Institute of Human Genetics, University of Jammu, Jammu and Kashmir (UT), 180006 India; 2 School of Biotechnology, University of Jammu, Jammu and Kashmir (UT), 180006 India; 3 Department of Zoology, University of Jammu, Jammu and Kashmir (UT), 180006 India

**Keywords:** Genetics, Zoology

## Abstract

Looking at the population’s behavior by taking samples is quite uncertain due to its big and dynamic structure and unimaginable variability. All quantitative sampling approaches aim to draw a representative sample from the population so that the results of the studying samples can then be generalized back to the population. The probability of detecting a true effect of a study largely depends on the sample size and if taking small samples will give lowers statistical power, higher risk of missing a meaningful underlying difference. The probability of rejecting the null hypothesis i.e., finding significant difference using the sample largely depends upon the statistical power. There are a lot of online tools used for calculating the sample size, but none tell us about the availability of samples from single site in a fixed span. This study aims to provide an efficient calculation method for the availability of samples during a specific period of a research study which is an important question to be answered during the research study design. So, we have designed a spreadsheet-based sample availability calculator tool implemented in MS-Excel 2007.

## Introduction

The transmission of genetic information from one generation to the next generation is a law of probability and population genetics take this concern to an entire population^1^. It makes us understand what are human variations, their origin, and their impacts on population by linking medical and evolutionary themes^2^. Apart from the “Clinical investigation” we pile up the facts related to the diseases by collecting history (assessment questionnaires) from individuals and establish the cause of a disease^3^ then estimates the individual risk of diseases and gives the chance of avoiding its risk of disease. This whole research study process is called epidemiological study which is also referred to as “population medicine”^4^. The epidemiological study is categorized under two different types i.e., Observational study and Experimental study. An observational study is further divided into three different classes including case–control study, cohort study, and cross-sectional study^5^. The retrospective study design determines whether exposure is associated with an outcome or not in a population by comparing two groups of matched cases and controls (Case–control study design)^6^ and establishes the risk factor of the diseases.

**Figure 1:**
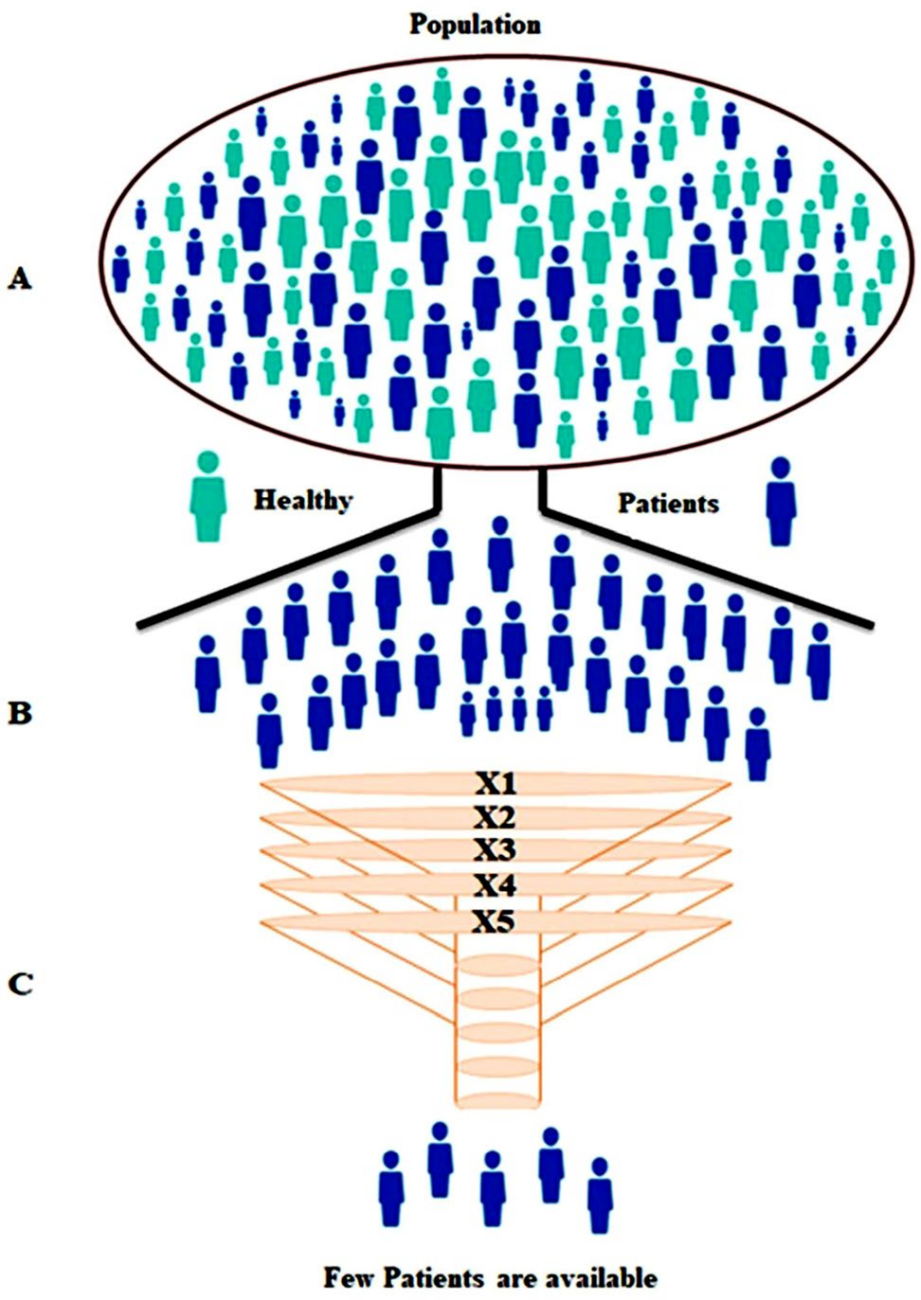
(**A**) Picture depicts a population having two kinds of individuals normal and diseased. (**B**) The number of patients is sorted out from the given population and then distributed into five different categories (**C**) Cases are distributed to five different categories (act as a filter) including those case who did not seek medical care, some case is from different geographical, some cases may be misdiagnosed, some may die and some may locate under unknown. Only small numbers of cases are available for the research study.

Population data are analyzed by different arms of science^7^ and used different terms to define the population. As per “biologist”, the number of all the organisms of the same group or species capable of interbreeding in a particular geographical area is called the population^8^. In this article we are strictly restricted to statistics, therefore, a population is an entire pool of people or events (hospital visits, small strata including clinics), from where fraction or percentage of a group is drawn which represents the statistical sample (Fig. 1A)^8^. Population, a big and dynamic structure with unimaginable variability, so looking at the population’s behavior by taking the whole population as a sample is quite uncertain and this is because of the restricted amount of time, ethical irrelevant, and money limitation. The quantitative sampling approach “quantifying the difference in effect, but unable to answer the question of *how it affects*”^9,10^ draws a representative sample through a random sampling approach from the considered population. The probability of success of a research study depends on the sufficient study sample size to produce clinically relevant difference^11,12^ but sometimes, not having a well-designed research study design tend to recruit a small sample size which increases the chance of assuming as true a false premise^13–15^. Having too large a sample size will become more expensive than necessary and also much time-consuming^16^. Studying with sample size calculations relates to the probability of a study correctly detecting a true effect^17^ to specify estimated parameters of the study design^18^.

There are a lot of online sample size calculators which are based on population size (https://www.calculator.net/sample-size; https://www.surveymonkey.com/mp/sample-size-calculator/; https://www.surveysystem.com/sscalc.htm; http://www.raosoft.com/samplesize.html; https://www.qualtrics.com/blog/calculating sample-size/), prevalence based (http://sampsize.sourceforge.net/iface/) and also on allele frequency (http://osse.bii.a-star.edu.sg/calculation1.php). Different from population-based studies is a hospital-based study^19^ which provides strata from where the patients were identified (a convenient based sampling)^20^ regardless of the population from which they arise^21^.

Despite having a huge population and a high incidence and prevalence, we never receive the requisite quantity of samples from a hospital. This is because individuals are avoiding medical treatment for a range of reasons, including unfavorable views of obtaining the medical treatment that includes factors related to doctors, ambulatory facilities, and emotional concerns, along with poorly perceived medical needs. Several individuals reported traditional barriers to medical care, such as high expenses, insurance, geographical barriers, sometimes there may be death or remission of patients before diagnosis^22^.

The tools listed above will inform us how many samples are needed for the study to find out the significant difference, but none of them will assist us to define a threshold for sample availability from a single hospital within a certain period. We attempted to tackle this difficulty in our work by using information gleaned from earlier data to generate predictions. The suggested model can be used to forecast what will happen/what will be the estimated number/sample/people that we will be able to obtain from a single hospital in a limited amount of time using the previous knowledge about population size and prevalence of the diseases. Therefore, this designed model is useful for calculating the probability of availability sample number per year and indicates that how much time we will need to complete the sampling.

Finding an exact number of patients/individuals/samples from a population is beyond the scope of the model. This model will help in setting a threshold for the availability of the sample from a single hospital and may inform about the exigency, which tells the researcher whether sampling needs to be done from the more than one hospital or region and thus serves as motivation for future research.

The remainder of the paper is laid out as follows: methods utilized in this study to design the algorithm/model, “Result” includes the result which represents the simulation data, “Discussion” represents the discussion and the conclusion of the study is present in “Conclusion”.

## Material method

**Figure 2:**
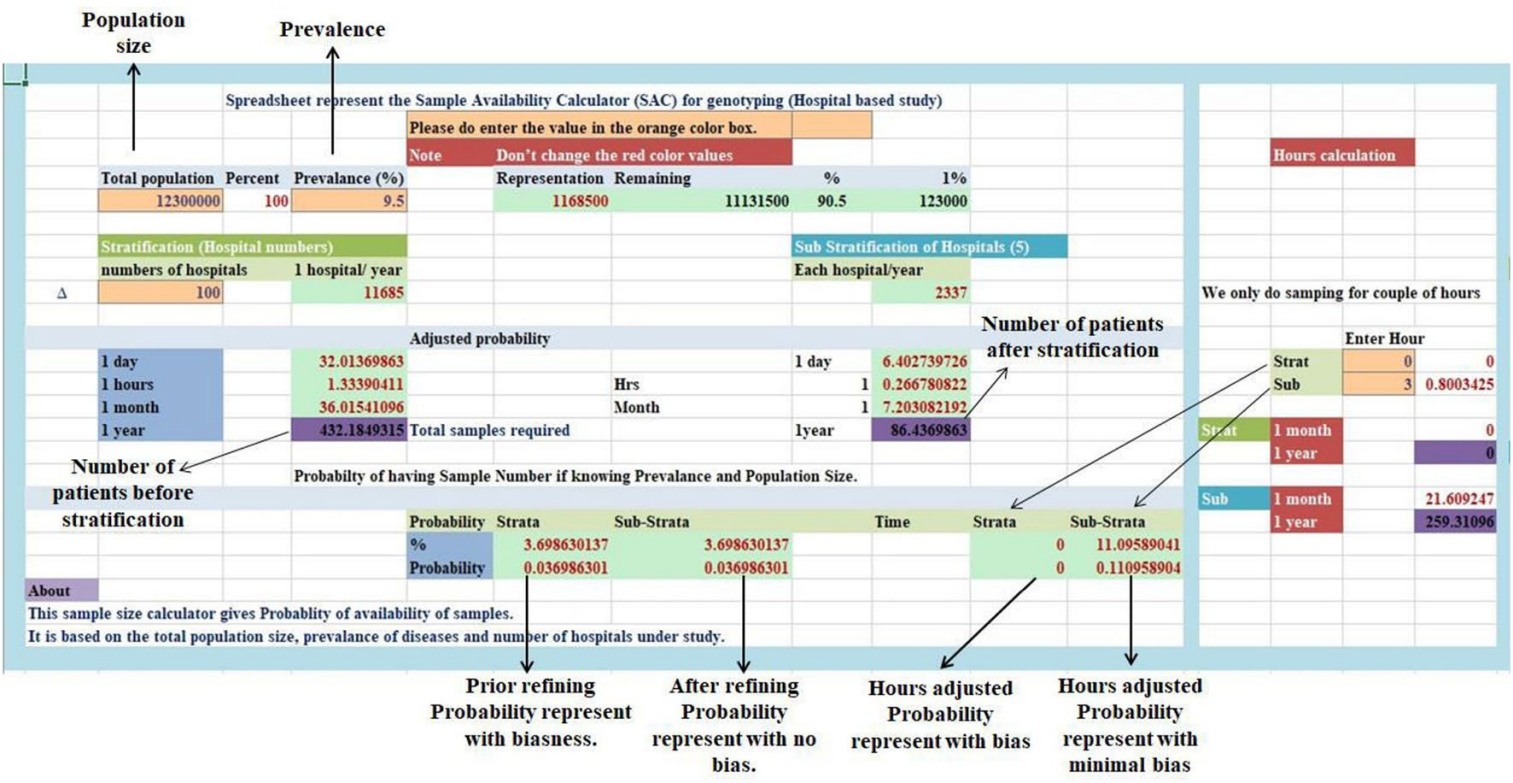
Main menu Sample availability calculator tool in MS-Excel 2002–2007.

In this research article, we are tried to solve a problem that we and most of the researchers faces during their pre-study design “estimating how much time will take to cover the required sample size”. This sample availability calculator based on the “probability” will set a threshold for the availability of the sample from a single hospital and is implemented in MS-Excel (Fig. 2) and can run on MS-Excel 2000–2007 on MS-Windows 2000, XP, Vista, and Windows 7 beta.

### Algorithm

An essential tool in statistics is the probability which measures, “how much chance that a given event will occur”^23^ and which have been significantly evolved for the last decades. To solve the problem, this mathematical model which is an algorithm-based (set of steps to solve the problem) expressed in the formula (symbolically to construct a relationship between given quantities) helps to link every value of a variable to the probability.

First, we will sort out the number of patients from the given population using the Eq. (1) where we use the previous knowledge of the prevalence of a disease and population size.

1 $$\varvec{S}=\varvec{n}\times \frac{\varvec{Pr}}{\varvec{Pc}}$$

where “**S**” representing the sample availability per year from the total population, “**n**” is the total number of population/population or size of the population, “**Pr**” prevalence of the diseases, and “**Pc”** is the percentage (100%) represents the whole population (Fig. 1A).

Once we find out the number of diseased individuals (from the previous data/published data), we do a uniform distribution (**U**), where samples are equally distributed to the default number of the hospital. The reason for choosing this much of hospital number as default is because for a big population there are at least a hundred hospitals. Its numbers can vary with different population sizes (positive correlation) and this change can be represented as (∆) from population to population. Therefore, this is managed by setting a threshold for hospital number (constant number) 100 (X = 100).

For equal distribution to “X”, Eq. (2) is used.

2 $${\varvec{U}}={\varvec{S}}\times \frac{1}{\Delta {\varvec{X}}}$$

where “U” is the uniform distribution of samples to a variable representing “X” and “S” is sample availability per year from the total population from Eq. (1).

As we know that despite having a huge population and a high incidence and prevalence, we never receive the requisite quantity of samples from a hospital and this is because of the following reasons^22^. Therefore, it is very important to introduce variables that may have an effect on the sample numbers and thus increasing the probability and overcome the bias that may be created during stratified (hospital) sampling. A variable that includes the number of cases who did not seek medical care or may some cases be seen elsewhere geographically, there may be death or remission of patients before diagnosis, etc. (Fig. 1C). Therefore, excluding these cases which may be responsible for creating bias and may affect the result, we equally distribute the stratified population into 5 different variables (X prime/ X'). Thus, dividing into smaller groups reduces variance and completes the sampling process.

3 $${{\varvec{S}}}^{\boldsymbol{^{\prime}}}={\varvec{U}}\times \frac{1}{{\varvec{X}}\boldsymbol{^{\prime}}}$$

where **S'** represents “sample available for sampling” after sub stratification in 5 different layers. Each variable representing cases that did not seek medical care (X'1), cases that are seen elsewhere geographically (X'2), cases misdiagnosed (X'3), death or remission before diagnosis (X'4), unknown variable (X'5). Selecting an unknown variable is to remove the biases created by a variable that canot be defined but may have an effect on our experimental data (confounding effect). After filtering from these only a few individuals are available for the case–control study. The reason for the equal distribution is that the chance of distribution and selecting samples will remain the same for all if we chose uneven distribution then we cannot say its probability because it will become “definitely/surely”. We need the “chance of outcome” not the “definitely it will be the outcome” because it is not applicable for so big a population which dynamic and changeable. By combining all the Eqs. (1, 2, and 3) we get,

4 $${\varvec{A}}={\varvec{n}}\times \frac{{\varvec{P}}{\varvec{r}}}{{\varvec{P}}{\varvec{c}}}\left(\frac{1}{\Delta {\varvec{X}}}\right)\left(\frac{1}{{{\varvec{X}}}^{\boldsymbol{^{\prime}}}}\right)$$

where “A” represents the availability of the sample per year at a particular hospital[after equally distributed to each variable (X)'].

**Figure 3:**
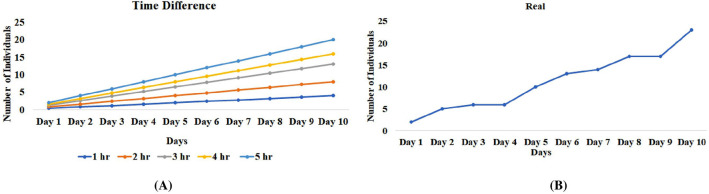
Time difference graphs (TDG): TDG shows that if we increase the time such as 1 h, 2–5 h. of the sampling there is an increase chance of getting more sample from the hospital. Therefore, represent a direct relationship of sample availability and time. In the second graph i.e., Real graph, which represent fluctuation in sample availability at each day, which reflect that we would never receive the exact number of samples as predicted by the model thus, represent the real-world diversity. But it helps us to set a threshold of sample availability from a single hospital with specific time span.

As we are sampling from the real-world situation where there is a limitation of works, time, patients, etc. so to overcome all these real-world situations we did some tricky calculations for the time and the day calculation which is important to reduce the chance of bias and so increasing the probability. For being with the smallest probability we chose 1-h representation, which means only we have limited access to the patients, and also, we exclude Sunday because OPDs (out patients department) are not open on Sunday.

5 $${\varvec{R}}=\frac{{\varvec{A}}}{{\varvec{Y}}{\varvec{d}}}\times \left(\frac{1}{{\varvec{H}}{\varvec{d}}}\times {\varvec{d}}{\varvec{M}}\times {\varvec{d}}{\varvec{Y}}\right)$$

where “**R**” is the value after refining, “**A**” is the availability of the sample per year at a particular hospital, “**Yd**” is the days in the year (365 days), “**Hd**” is hours in the days (24), “**dM**” is days in the month [excluding Sunday (26), and “**dY**” is the total month in a year (12)]. It is important to note that if we increase the time more patients will come and thus the chance of getting patients will also increase (Fig. 3B).

The probability of sample availability per year is calculated by using Eq. (6),

6 $${\varvec{P}}=\frac{{\varvec{R}}}{{\varvec{A}}}\left(\frac{{\varvec{P}}{\varvec{c}}}{100}\right)$$

where “P” is the probability of availability of sample per year, “R” is the data value after refining, “A” is the availability of the sample per year at a particular hospital, and “Pc” is the percentage (100%). Probability for 1 day with time managed (1 h to 10).

## Result

Here, to check the effectiveness of the model first we used the model on the imaginary data or with random numbers, where we took a population size of about 58,746,995 with the varied prevalence of imaginary diseases including 3%, 9%, 10%, 12%, 15%, and, 17%. After using the model for calculating the number of available samples, we found that there are approximately 651, 1955, 2172, 2607, 3259, and 3693 individuals (before refining). In a real situation, we will never get so many samples because of several reasons (discussed above in the introduction) thus, the number deviates from the calculated samples. Therefore, it is important to introduce the variables (refining) and also equal distribution of cases in these five different categories which will result in minimum bias. Thus, after introducing the variables we found 130, 391, 434, 521, 651, and 738 numbers of individuals which was much different from the previous calculation so it's important to do refining with equal distribution.

We also tested the model by adjusting the timing, for example taking the above population size i.e., 58,746,995 individuals with the prevalence rate of 3% with 1 h gives 651 individuals (before refining) in one year of span. If we increase the time from 1 to 2 h and 3 h. there are about 1304 and 1956 individuals respectively and so on. But as we now know that without refining there may be a high chance of bias so after refining population size of 58,746,995 with the prevalence rate of 3% with 1 h gives 130 individuals and increasing hour represent 260, 391 individuals and so on in one year (repeat the test with all the imaginary data numbers listed in the above section) (Fig. 3).

**Figure 4:**
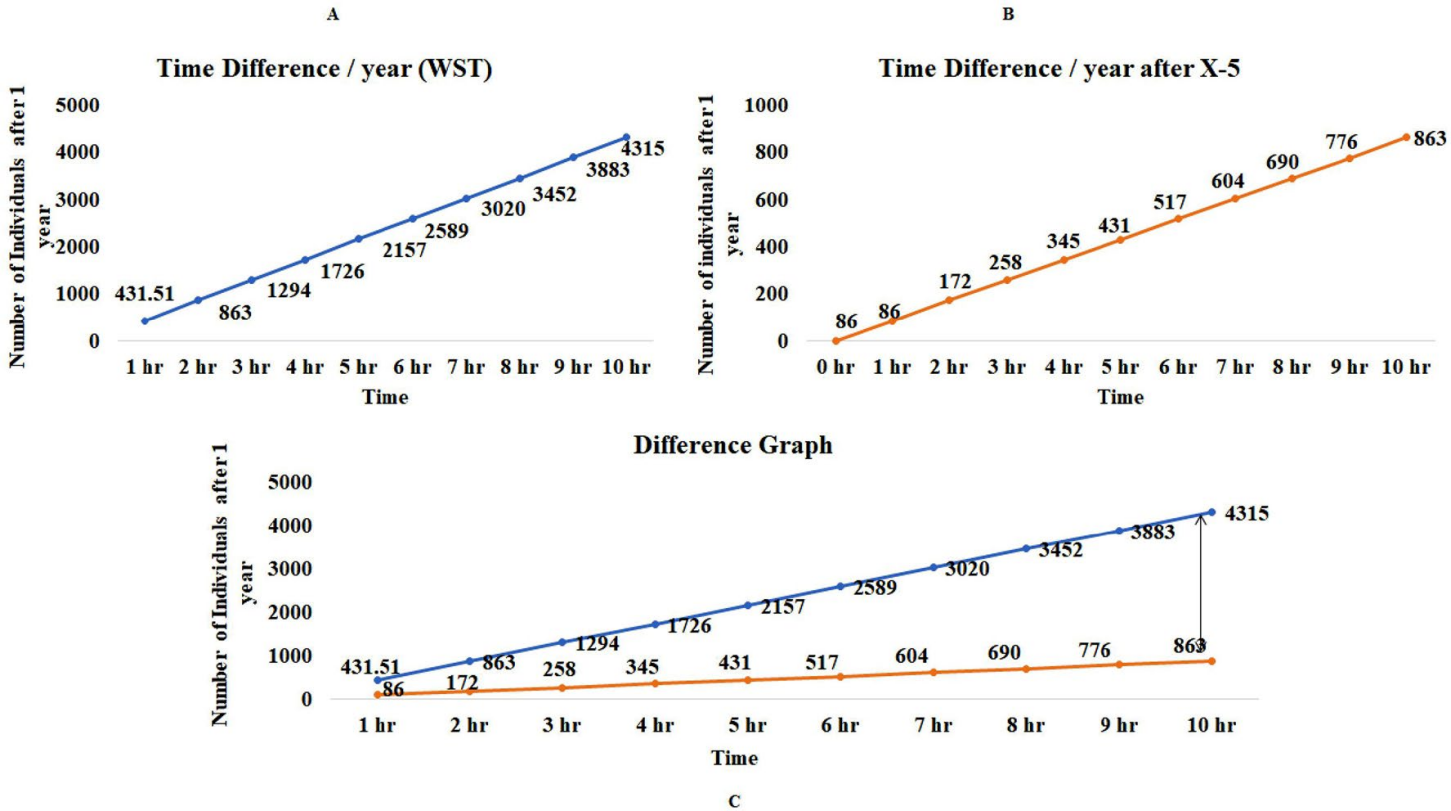
(**A**) The WST graph depicts the number of available samples before introduction of variable, as we increase the time of sample collection, the chances of getting individuals will also increase and the probability of getting samples will increase. (**B**) Graphs depict the availability of samples per hospital after introduction of variable and time adjustment: if we increasing the time the chance of getting individuals also increased. (**C**) Difference graph shows how the introduction of variable minimize the bias (*WST* without stratification).

The best representation of time difference is well presented in **(**Fig. 4). Here using 1 h per day represent the lowest probability and maximum probability will be directly proportional to the maximum hours of the day (Fig. 4A). After the introduction of variable and time adjustment (Fig. 4B), we can see there is a lot of difference (Fig. 4C) and provide an estimate of sample availability.

**Figure 5:**
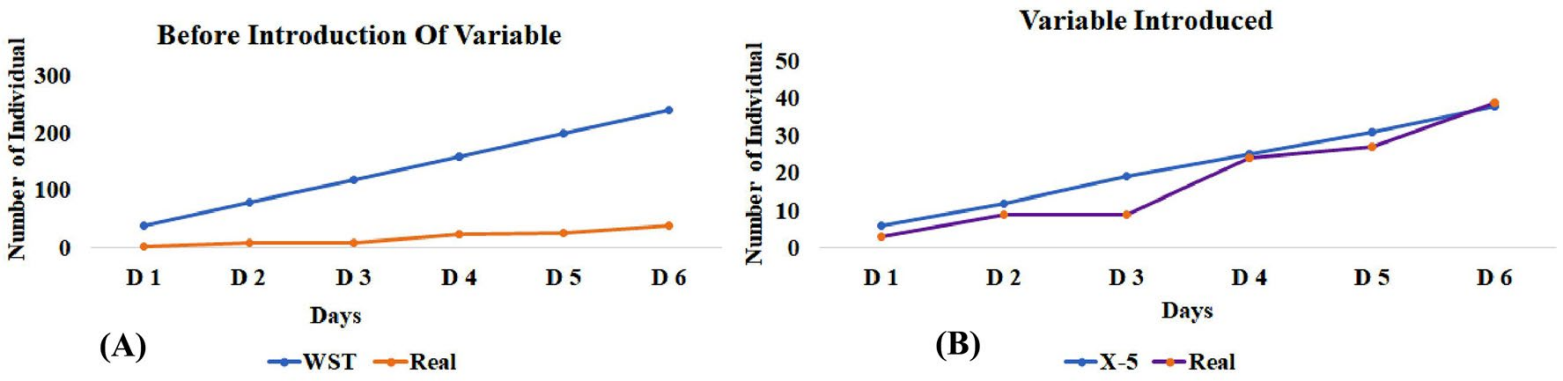
(**A**) Graph representing the number of individuals (Y-axis) found at each day (X-axis) with 1-h adjustment. Blue graph line indicates the number of individuals that will be available. In the real situation, we will never get so many samples because of individuals are avoiding medical treatment for a range of reasons thus the sample number deviate from the calculated samples. (**B**) Introduction of variables (X1, X2, X3, X4, X5) and uniform distribution of patients in these five different categories leads to minimum bias (*WST* without stratification).

Second, apart from simulated data, we tested the model's efficacy in our population (Jammu and Kashmir-north Indian population) of 1.23 crore people (https://www.populationu.com/in/jammu-and-kashmir-population) with a specific condition, migraine, which has a prevalence rate of around 12%^24^. The model predicts the total availability of approximately 109 samples (with 1 h.) and with an increase in the time limit from 1–5 h, the availability of samples also increases (218, 327, 436, and 545 samples). After completion of the year (sampling period), we found around 380 samples with 3 h each day at OPD. So, we saw here both similarities (crosses the threshold of 327) (Fig. 5) as well as the difference of around 53 samples this may be due to the involvement of high diversification reasons^22^.

Also, we checked it on another disease i.e., Kidney stone (nephrolithiasis) with a prevalence rate of 15%^25^ in the same population, and approximately 409 samples were predicted with 3 h. time limit. After completion of the sampling period i.e., 1 year, we found around 480 samples with 3 h each day at OPD. We also checked it on the pre-studied condition from our labs such as breast cancer which have a prevalence rate of 6% (https://www.cancer.net/cancer-types/breast-cancer/statistics), leukemia (9.5)^26,27^ and Coronary artery diseases (CAD) (16%)^28^ has found around 60^29^ 210 patients^30^ and 400 subjects per year^31^ respectively which is near to the calculated numbers by the model and also on congenital heart diseases (CHD) (8%)^32^ and found 80 subjects per year (not published yet).

To this end, we would never receive that many samples in the actual world, and the sample number differ from the estimated samples as shown in the graphs (Fig. 5B). Therefore, this model will assist in determining a sample availability threshold from a single hospital, as well as information on the urgency, which will notify the researcher whether sampling from more than one hospital or area is required.

## Discussion

In epidemiology studies, the most frequent type i.e., case–control studies (a retrospectives study) is used to determine that exposure is associated with an outcome (i.e., disease or condition of interest) or not, and^6^. In a population-based case–control study, cases are ascertained from a disease registry or from hospital networks from a specific geographical area within a specified period^33^ to study the associate risk factor and estimate the effect of exposure on the risk of diseases.

But the question is how many numbers of samples from the population are required to draw out the meaningful difference? and the probability of detecting a true effect of a study for a population that is very dynamic with unimaginable variability largely depends on the sample size. If we take a small sample size which will give lowers statistical power, higher risk of missing a meaningful underlying difference. Here biomedical statistics have come under increased scrutiny^11^.

There are a lot of online sample size calculators which are based on population size, prevalence based, and also on allele frequency which tell us about the number of samples required for the research study to find out the significant difference. But none tool will help in setting a threshold for the availability of the sample from a single hospital in a particular period.

A well-designed spreadsheet in MS-Excel 2000–2007 will help in the calculation which is set accordingly to the algorithms that are stated above. It can run on MS-Excel 2000–2007 on MS-Windows 2000, XP, Vista, and Windows 7 beta. We just have to enter the total population size, the prevalence, the total hospital will remain to the defaults if want to change its editable, all these will provide the exactly equally distributed samples accordingly to the time mentioned. The sample availability tool in MS-Excel is readily available to any researcher and wishes to use it for non-commercial purposes without any restriction.

Finding an exact number of patients/individuals/samples from a population is beyond the scope of the model. This model will assist in determining the sample availability threshold from a single hospital, as well as information on the urgency, which will notify the researcher whether sampling from more than one hospital or area is required, and therefore act as encouragement for future study.

## Conclusion

This sample availability calculation tool will help in finding the number of samples that are available during the specific period of your research study and thus meet your required sample size to detect absolute power. This sample availability calculation is well-designed in an excel spreadsheet (MS-Excel 2000–2007) (Fig. 2) which can run on MS-Excel 2000–2007 on MS-Windows 2000, XP, Vista, and Windows 7 beta and will use it for non-commercial purposes without any restriction and act as encouragement for future study.

## Supplementary Information

Supplementary Information.
